# Online counseling experience of Turkish counselor candidates throughout COVID-19 pandemic

**DOI:** 10.1192/j.eurpsy.2021.1779

**Published:** 2021-08-13

**Authors:** H. Korkmaz, B. Güloğlu

**Affiliations:** 1 Psychological Counseling And Guidance, Bahçeşehir University, Beşiktaş/İstanbul, Turkey; 2 Psychological Counseling, Bahcesehir University, Istanbul, Turkey

**Keywords:** online counseling, COVID-19, Turkish Counselor Candidates, Therapeutic relationship

## Abstract

**Introduction:**

As in many areas of life, the covid-19 epidemic has had a great impact on psychological counselor training. Although studies and practices on online counseling are increasing every day in the world, there has not been a psychological counseling method preferred by experts in Turkey, which comes from community culture and, where physical contact is important, until the pandemic.

**Objectives:**

The examination of the opinions of the students studying in the last year of the psychological counseling and guidance undergraduate program during the pandemic regarding online counseling, where they perform their first psychological counseling experience.

**Methods:**

The study was conducted with 10 counseling students, 9 women and 1 Man. The age range of the students is 22-24 and the average age is 20.6. The students’ opinions are taken with open-ended questions such as “Can you share your views on online counseling before online counseling?“ The reflection letter that the students responded to was subjected to content analysis.

**Results:**

The findings of the study show that there are four themes: Emotions before the counseling process, Thoughts before the counseling process, Therapeutic relationship, Online counseling in professional life. For example; in online counseling, negative emotions such as anxiety, excitement, fear, anxiety, stress, anxiety, as well as feeling comfortable and safe are among the positive feelings they experience in their therapeutic relationships.

**Conclusions:**

As a result, although students have a positive view of online counseling, they mainly prefer to do it face-to-face. The findings were discussed taking into account Turkish culture.

**Disclosure:**

No significant relationships.

